# Absolute expressions of hypoxia-inducible factor-1 alpha (*HIF1A*) transcript and the associated genes in chicken skeletal muscle with white striping and wooden breast myopathies

**DOI:** 10.1371/journal.pone.0220904

**Published:** 2019-08-08

**Authors:** Yuwares Malila, Krittaporn Thanatsang, Sopacha Arayamethakorn, Tanaporn Uengwetwanit, Yanee Srimarut, Massimiliano Petracci, Gale M. Strasburg, Wanilada Rungrassamee, Wonnop Visessanguan

**Affiliations:** 1 National Center for Genetic Engineering and Biotechnology (BIOTEC), Khlong Nueng, Khlong Luang, Pathum Thani, Thailand; 2 Department of Agricultural and Food Sciences, Alma Mater Studiorum, University of Bologna, Cesena (FC), Italy; 3 Department of Food Science and Human Nutrition, Michigan State University, East Lansing, MI, United States of America; Heidelberg University, GERMANY

## Abstract

Development of white striping (WS) and wooden breast (WB) in broiler breast meat have been linked to hypoxia, but their etiologies are not fully understood. This study aimed at investigating absolute expression of hypoxia-inducible factor-1 alpha subunit (*HIF1A*) and genes involved in stress responses and muscle repair using a droplet digital polymerase chain reaction. Total RNA was isolated from pectoralis major collected from male 6-week-old medium (carcass weight ≤ 2.5 kg) and heavy (carcass weight > 2.5 kg) broilers. Samples were classified as “non-defective” (n = 4), “medium-WS” (n = 6), “heavy-WS” (n = 7) and “heavy-WS+WB” (n = 3) based on abnormality scores. The *HIF1A* transcript was up-regulated in all of the abnormal groups. Transcript abundances of genes encoding 6-phosphofructo-2-kinase/fructose-2,6-biphosphatase 4 (*PFKFB4*), lactate dehydrogenase-A (*LDHA*), and phosphorylase kinase beta subunit (*PHKB*) were increased in heavy-WS but decreased in heavy-WS+WB. Glyceraldehyde-3-phosphate dehydrogenase (*GAPDH*) was up-regulated in non-defective samples. The muscle-specific mu-2 isoform of glutathione S-transferases (*GSTM2*) was up-regulated in the abnormal samples, particularly in the heavy groups. The genes encoding myogenic differentiation (*MYOD1*) and myosin light chain kinase (*MYLK*) exhibited similar expression pattern, of which medium-WS and heavy-WS significantly increased compared to non-defective whereas expression in heavy-WS+WB was not different from either non-defective or WS-affected group. The greatest and the lowest levels of calpain-3 (*CAPN3*) and delta-sarcoglycan (*SCGD*) were observed in heavy-WS and heavy-WS+WB, respectively. Based on micrographs, the abnormal muscles primarily comprised fibers with cross-sectional areas ranging from 2,000 to 3,000 μm^2^. Despite induced glycolysis at the transcriptional level, lower stored glycogen in the abnormal muscles corresponded with the reduced lactate and higher pH within their meats. The findings support hypoxia within the abnormal breasts, potentially associated with oversized muscle fibers. Between WS and WB, divergent glucose metabolism, cellular detoxification and myoregeneration at the transcriptional level could be anticipated.

## Introduction

An artificial selection program for fast growing broilers has been developed over the recent decades and implemented in response to consumers’ rising demand for chicken meat [1]. The growth period of broilers from hatch to market-age has been reduced by half while the body weight at market-age has doubled compared to broilers of 70 years ago [2, 3]. Concomitant with the accelerated growth rate, occurrences of abnormalities, especially in breast muscles, have been consistently reported [4, 5].

White striping (WS) and wooden breast (WB) have been the two emerging meat abnormalities provoking wide concern. Despite similar histological lesions including necrosis and myodegeneration, inflammation, mononuclear cell accumulation, fibrosis, and lipidosis [6–9], the two myopathies exhibit different phenotypic characteristics. The WS syndrome is recognized by the appearance of white striations on the meat surface parallel to the direction of muscle fibers whereas WB is characterized by development of diffuse or focal rigidity within the superficial breast muscles [4, 7, 10]. The WS abnormality was first reported in 2012 with a prevalence of 12% per flock [5] but its incidence has increased remarkably to almost 50% in 2014 [11] and over 90% during 2017 [12] and 2018 [13]. Development of the WS abnormality not only adversely affects consumers’ acceptance [14], but also impairs the nutritional quality and technological properties of the broiler meat [13, 15–17]. On the other hand, the incidence of WB has usually been found at approximately 6.5% [13], but the impact of this condition is much more deleterious compared to WS meat [13].

Although the exact causes of the WS and WB abnormalities are still not fully understood, growing evidence suggests a possible link to growth-induced myopathies [7, 18–20]. The hypertrophied muscle fibers occupy areas originally maintained by connective tissue networks, reducing spaces for capillaries [21–23]. An insufficient vascularization reduces the efficiency of waste removal, leading to an accumulation of metabolic end products that could induce oxidative stress and eventually leading to necrosis within the muscle [24, 25]. Under hypoxic conditions, muscle regeneration could be impaired, leading to development of WS or WB abnormalities or both. Previous -omics studies at the levels of transcripts [24, 26, 27], proteins [28] and metabolites [29] have shown that hypoxia within a breast muscle is a likely cause of WS and WB developments in broilers [29].

In response to hypoxic stress, the hypoxia-inducible factor-1 (HIF-1) protein, a heterodimeric protein comprising HIF-1α and HIF-1β subunits [30], plays a major role in mediating adaptive mechanisms. When cellular oxygen becomes limited, HIF-1α protein level increases [31] and the protein translocates into the nucleus to form an active complex with the HIF‐1β subunit. The activated HIF‐1 complex induces transcription of hypoxia-responsive elements involved in several biological pathways such as glycolysis, glycogen and lipid metabolism [32]. By exploiting the Illumina RNA sequencing technique, Mutryn et al. [24] compared transcriptome profiles of pectoralis major muscle with or without WB myopathy and identified differential expression of several HIF-responsive genes. Using a similar platform, Marchesi et al. [27] recently observed an increased expression of *HIF1A* in the WS muscle samples. Based on those two studies, development of WS and WB abnormalities were suggested to be the responsive processes against hypoxia and cellular stress.

Among those hypoxia-responsive pathways, the switch of energy metabolism from oxidative phosphorylation to glycolysis is one of the responsive mechanisms which not only reserves cellular oxygen and energy but also prevents an excessive formation of detrimental reactive oxygen species (ROS) [33]. However, an increased accumulation of ROS under hypoxia usually occurs as a consequence of impaired mitochondrial function and limited vascularization, leading to the accumulation of some harmful intermediate metabolic products [34, 35]. To counter the oxidative stress, antioxidant and detoxification mechanisms, particularly glutathione S-transferase (GST) superfamily, are up-regulated [36, 37]. Under such stress, muscle cells can undergo degeneration; thus, the cascade of regeneration processes is subsequently triggered [38]. However, the understanding regarding stress-response mechanisms, particularly via the HIF-1 induction, of both WS and WB abnormalities still needs to be established.

The objective of this study was to investigate the absolute expression level of *HIF1A* in association with development of WS and WB myopathies in chicken breast muscle. Ten additional key genes representing relevant responsive functions including glucose utilization, antioxidation, and myoregeneration, were also included in the analysis. Absolute transcript abundances of the target genes were quantified using a droplet digital polymerase chain reaction (ddPCR). Unlike the previous generations of PCR-based techniques, ddPCR, labeled as the 3^rd^ generation of PCR technique, was developed for quantifying absolute copy numbers of initial target molecules. Each ddPCR reaction mixture is partitioned into up to 20,000 water-in-oil droplets in which the templates are randomly distributed. The presence or absence of target templates in each droplet is detected by fluorescence and designated as a positive or negative droplet, respectively. The fractions of positive and negative droplets are automatically fit based on a Poisson algorithm to determine the initial concentration of target molecules. Overall, this technique enables determination of the concentration of starting molecules without preparation of a standard curve or identification of any reference genes.

## Materials and methods

### Sample collection and WS classification

The breast specimens used in this study were from the same sample group previously described in the study of Malila et al. [13]. In brief, the carcasses of male Ross 308 broilers, raised at a facility of a local poultry company to six weeks of age and processed in the industrial processing line under the routine Halal standard practice, were supplied from a local slaughterhouse (Sara Buri, Thailand). Immediately after the defeathering step, the pre-rigor whole carcasses were randomly collected from processing conveyer and proceeded to the research operation station. The station was set up nearby the processing line to ensure the integrity of the muscle and RNA materials. The breast muscles were immediately removed from the left side of each supplied carcass, cut into 1 cm × 1 cm × 1 cm cubes, snap frozen in liquid nitrogen and stored in liquid nitrogen while transported back to the Food Biotechnology Laboratory, National Center for Genetic Engineering and Biotechnology (BIOTEC) located in Pathum Thani (Thailand). Immediately upon arrival, the muscle samples were stored in a freezer at -80°C until used for RNA isolation and for determination of pH at 20-min postmortem (pH_20m_), ATP status, glycogen (Gly_20m_) and lactate content (Lac_20m_). The breast from the right side of the chicken was removed from the carcass, individually packed in a plastic bag, and stored on ice during the transportation back to the laboratory. Upon arrival, the breasts were stored at 4°C until they reached 24-h postmortem. The samples, labeled as 24-h postmortem meats were subsequently used for classifying meat degree of abnormality as well as pH at 24-h postmortem (pH_24h_) and lactate concentration (Lac_24h_).

In this study, all samples were purchased in the form of whole carcasses from the commercial processing plant. Either experimental treatments or scientific procedures were subjected to the living animals. Thereby, according to BIOTEC Institutional Animal Care and Use Committee, the ethical approval was not required.

It is worth noting that upon the sample collection, only four non-defective breasts were found among the samples. Based on our initial objectives to compare absolute gene expression between non-defective and defective samples, 13 samples exhibiting moderate WS condition (40 white lines with ≤ 0.5 mm thickness or 1–5 lines with 1–1.9 mm thickness) were randomly collected from either the group of broilers with carcass weight less than or equal 2.5 kg (“medium”, n = 7) or the group with carcass weight greater than 2.5 kg (“heavy”, n = 6). The final group of samples consisted of three heavy broilers exhibiting both WS and WB abnormalities classified as described elsewhere [13]. Accordingly, there were 20 samples classified into four sample groups for further analysis: “non-defective” (n = 4), “medium-WS” (n = 7), “heavy-WS” (n = 6) and “heavy-WS+WB” (n = 3).

### RNA isolation and cDNA synthesis

Total RNA was isolated from the frozen breast muscle samples using TRIzol Reagent (Invitrogen) according to manufacturer’s protocol. Contaminating genomic DNA was eliminated using DNase I (Thermo Scientific, Inc.) and the isolated RNA was re-purified using a GeneJet RNA Cleanup and Concentration Micro kit (Thermo Scientific, Inc). Quantity and quality of total RNA were determined using a Nanodrop spectrophotometer (Thermo Scientific, Inc.) and an Agilent Bioanalyzer model 2100 (Agilent Technologies, Inc.), respectively. Only RNA samples exhibiting RNA integrity number (RIN value) greater than 7.5 were used for cDNA synthesis. The cDNA was synthesized from 1.5 μg of total RNA with oligo(dT) primer using ImPromII Reverse Transcription System kit (Promega). The amount of the synthesized cDNA was determined using a Nanodrop spectrophotometer (Thermo Scientific, Inc.).

### Primers

Absolute transcript abundances were determined for eleven target genes including *HIF1A*, 6-phosphofructo-2-kinase/fructose-2,6-bisphosphatase-4 (*PFKFB4*), lactate dehydrogenase A (*LDHA*), phosphorylase kinase, beta (*PHKB*), glyceraldehyde-3-phosphate dehydrogenase (*GAPDH*), glutathione S-transferase alpha 4 (*GSTA4*), glutathione S-transferase mu 2 (*GSTM2*), myogenic differentiation 1 (*MYOD1*), calpain 3 (*CAPN3*), sarcoglycan, delta (*SCGD*) and smooth-muscle isoform of myosin light chain kinase (*MYLK*). Of these eleven genes, *PFKFB4*, *LDHA*, *PHKB*, and *GAPDH* encode key enzymes involved in glucose utilization. The enzymes encoded by *GSTA4* and *GSTM2* are in the GST superfamily which are responsible for detoxification by neutralizing toxic compounds which can be easily removed from the cells to reduce oxidative damage. The other four play essential roles in muscle repair processes and are associated with development of muscle myopathies. Further details on the roles of these genes are discussed in the results and discussion section.

Reference sequences from The National Center for Biotechnology Information (NCBI) were used as template for primer design. All primers were designed using Primer-BLAST (https://www.ncbi.nlm.nih.gov/tools/primer-blast/) and are listed in Table 1. Amplicon sizes were limited to fewer than 200 bp as recommended for the ddPCR assay. OligoAnalyzer (https://www.idtdna.com/calc/analyzer) was used for secondary structure and dimer prediction. Only primers that matched recommended criteria (GC content 40–60%, melting temperature 50–65°C, ΔG > -5) were synthesized by Macrogen (Korea).

**Table 1:** Primers designed for EVAGREEN-based droplet digital polymerase chain reaction.

NCBI Accession	Gene ID	Gene annotation	Sequence (5’→3’) (F: forward, R: reverse)	Amplicon Size (bp)	Template quantity (ng/20 μL reaction)
NM_001004405.2	*CAPN3*	Calpain 3	F: GAACAACCAGCTCTACGACA R: TGGAATGCCCTGAACATAGC	117	10
NM_204305.1	*GAPDH*	Glyceraldehyde-3-phosphate dehydrogenase	F: ACTTTGGCATTGTGGAGGGT R: GGACGCTGGGATGATGTTCT	131	1
XM_015284816.1	*GSTA4*	Glutathione S-transferase alpha 4	F: TGCCACTGGTTGAGATCGACG R: TCTCCTTTGCCTCAGGTGGA	192	100
NM_205090.1	*GSTM2*	Glutathione S-transferase mu 2	F: GTGGACTTCCTGGCTTACGA R: GCCGTGTACCAGAAAATGG	173	10
XR_001466725.2	*HIF1A*	Hypoxia inducible factor 1, alpha subunit	F: ATCAGAGTGGTTGTCCAGCAG R: CAGTCCAAGCCCACCTTACT	111	25
NM_205284.1	*LDHA*	Lactate dehydrogenase A	F: TTCTCTGCCAGCTGAATAGCTT R: CGGGTCATTGTCTTGTTGCAT	200	1
AH_006335.2	*MYLK*	Myosin light chain kinase (smooth muscle isoform)	F: ACAGACTGAAAGCACAAAGACAG R: CAGAGCCATTCACTGACGGT	171	100
NM_204214.2	*MYOD1*	Myogenic differentiation 1	F: AGGAAACCTGAGTGACAGTGG R: GACCTGCCTTTATAGCACTTGG	121	10
XM_015273127.1	*PFKFB4*	6-phosphofructo-2-kinase/fructose-2,6-biphosphatase 4	F: ATGCTACAAAGCCACCTACG R: GTGTGTGTTCATCAGGTAGTAC	151	10
NM_001007831.1	*PHKB*	Phosphorylase kinase, beta	F: GCACGGTGTAGTAATTGTTGC R: GGGCACTTTGTGTCTCTAATG	148	10
XM_015293901.1	*SCGD*	Sarcoglycan, delta	F: CCTGAAGCCTCATACAGCAA R: TATTCTTCTGCTCCACAGCG	145	10

### Absolute expression analysis

The synthesized cDNA was used as a template for an EVAGREEN-ddPCR system. The 20-μL reaction contained 1X EVAGREEN supermix (Bio-Rad), 0.25 μM of each forward and reverse primer and cDNA template of 1–100 ng/reaction. The concentration of the templates for each primer was optimized and are shown in Table 1. No template control (NTC) was added in every run by replacing the cDNA sample with an equal amount of nuclease-free water. The droplet emulsion was generated by a QX100 droplet generator (Bio-Rad) according to Bio-Rad’s instruction. The droplets were transferred to 96-well plate and amplified using a conventional thermocycler (T100 thermal cycler, Bio-Rad). The cycling condition was as follows: enzyme activation at 95°C for 5 min, 40 cycles of denaturation at 95°C for 30 s and annealing/extension at 58°C for 1 min, followed by signal stabilization at 4°C for 5 min and 90°C for 5 min. After PCR, the fluorescent signal intensity of the droplets was measured using a QX200 droplet reader (Bio-Rad). The detected droplets were depicted in the format of one-dimensional graph. The threshold line was set under the high amplitude droplet cluster to separate positive and negative droplets and to avoid false positive from non-target amplicons (droplet rains). The initial concentration of targets was calculated by a QuantaSoft droplet reader software (Bio-Rad) and expressed in copies per 20 μL reaction and further calculated to copies per nanogram template.

### Determination of pH and ATP status

The pH_20m_ was measured using the method described by Eadmusik et al. [39] with a slight modification. In brief, 1.0 g of the snap-frozen sample was homogenized in 10 mL iodoacetate buffer (5 mM sodium iodoacetate, 150 mM potassium chloride, pH 7) for 30 s. The pH of the homogenate was determined using a pH meter (Mettler-Toledo Seven Easy, Mettler-Toledo, Inc., Switzerland). For pH_24h_, the pH was directly measured by inserting a glass pH-probe (Mettler-Toledo, Inc.) into three designated positions of each breast sample.

The ATP status of each snap-frozen muscle samples was evaluated using the R-value method as described by Ryu and Kim [40] with minor modifications. Briefly, 500 mg of the frozen sample was homogenized in 5 mL of 1 M perchloric acid for 40 s. The homogenate was centrifuged (Eppendorf 5810R, Eppendorf, Germany) at 5,000*×*g for 10 min, 4°C. One hundred microliters of the supernatant were mixed with 2 mL of 0.1 M phosphate buffer (pH 7.0). Absorbances at 250 nm and 260 nm of the mixture were subsequently measured using a UV-Vis spectrophotometer (Model Helios omega, Thermo Scientific, Inc.). The measurement was done in duplicate for each sample. The R-value was expressed as a ratio of the absorbance at 250 nm to absorbance at 260 nm (A_250_/A_260_).

### Glycogen assay

The Gly_20m_ was determined using a glycogen assay kit (Sigma-Aldrich, MO) following the company’s recommendation. Prior to the development of the colorimetric reaction, crude glycogen in the frozen muscle was extracted as previously described [41]. Briefly, 80 mg of the frozen tissue was heated in 300 μL of 30% KOH at 100°C for 2 h. Afterwards, 3 volumes of 95% ethanol were added to precipitate crude glycogen. The mixture was centrifuged at 3,000 rpm for 10 min. The resulting pellet was resuspended in 150 μL deionized water and acidified to pH 3 using HCl. The extracted glycogen was re-precipitated in 95% ethanol twice to remove any impurities. The final pellet was air-dried and subsequently dissolved in 300 μL deionized water. The measurement was conducted in duplicate. Muscle glycogen was expressed in milligrams per gram muscle sample.

### Lactic acid assay

Five hundred milligrams of either 20-min postmortem muscle or 24-h postmortem meat samples were homogenized with 10 mL of 1 M perchloric acid for 2 min. The pH of the homogenate was adjusted to pH 8.0 using KOH followed by volume adjustment to 25 mL using distilled water. The solution was then incubated on ice for 20 min to precipitate potassium perchlorate and subsequently centrifuged at 13,000×g for 10 min. Lactic acid content in the supernatant was determined using L-lactic acid assay kit (Megazyme, Ireland). The measurement was done in duplicate. Lactate in the sample was expressed in milligrams per gram muscle sample.

### Microscopic images

Cross-sectional microscopic views of the samples were imaged using an SU5000 field emission-scanning electron microscope (FE-SEM, HITACHI Ltd., Tokyo, Japan) according to the method of Wattanachant et al. [42] with modifications. The muscle samples (5 × 5 × 5 mm) were fixed in 2.5% glutaraldehyde, 0.1 M phosphate buffer, pH 7.3 for 2 h at ambient temperature. After washing with 0.1 M phosphate buffer, pH 7.3, the samples were serially dehydrated in ethanol solutions of 50%, 70%, 80%, 90% and 100%. The samples were dehydrated twice for 30 minutes each for the 50% - 90% ethanol solutions and three times for 30 min using 100% ethanol. The specimens were cut in liquid nitrogen, mounted on an aluminum stub using carbon tape and sputter-coated with gold for 15 sec. Cross-sectional images (300× magnification) of the samples were displayed under FE-SEM using an acceleration voltage of 10 kV and subsequently analyzed using WinROOF software (Mitani Corporation, Japan). The microscopic images were processed using ImageJ 1.46r software [43]. The size distribution of the fibers was expressed as a percentage relative to total number of fibers within each WS severity [44]. Fibers were grouped into four subdivisions; group I (<1,000 μm^2^), group II (≥1,000 μm^2^ to <2,000 μm^2^), group III (≥2,000 μm^2^ to <3,000 μm^2^) and group IV (≥3,000 μm^2^) based on individual cross-sectional area.

### Statistical analysis

Statistical analysis was conducted with R package version 3.2.1. One-way analysis of variance (ANOVA) was used to evaluate differences in means among non-defective and the other abnormal samples. The groups of data with significant differences in means (p<0.05) were separated using Duncan’s new multiple range test. Prior the analysis of ANOVA, Shapiro-Wilk Normality Test and Bartlett Test were conducted to monitor normal distribution and homogeneity of variance, respectively, of the dataset. The dataset that failed to follow the assumptions were transformed before re-subjected to ANOVA and multiple range test. The significance level for all statistical analyses was set as α = 0.05.

## Results and discussion

### Muscle fiber size distribution in abnormal meat samples

**Fig 1:**
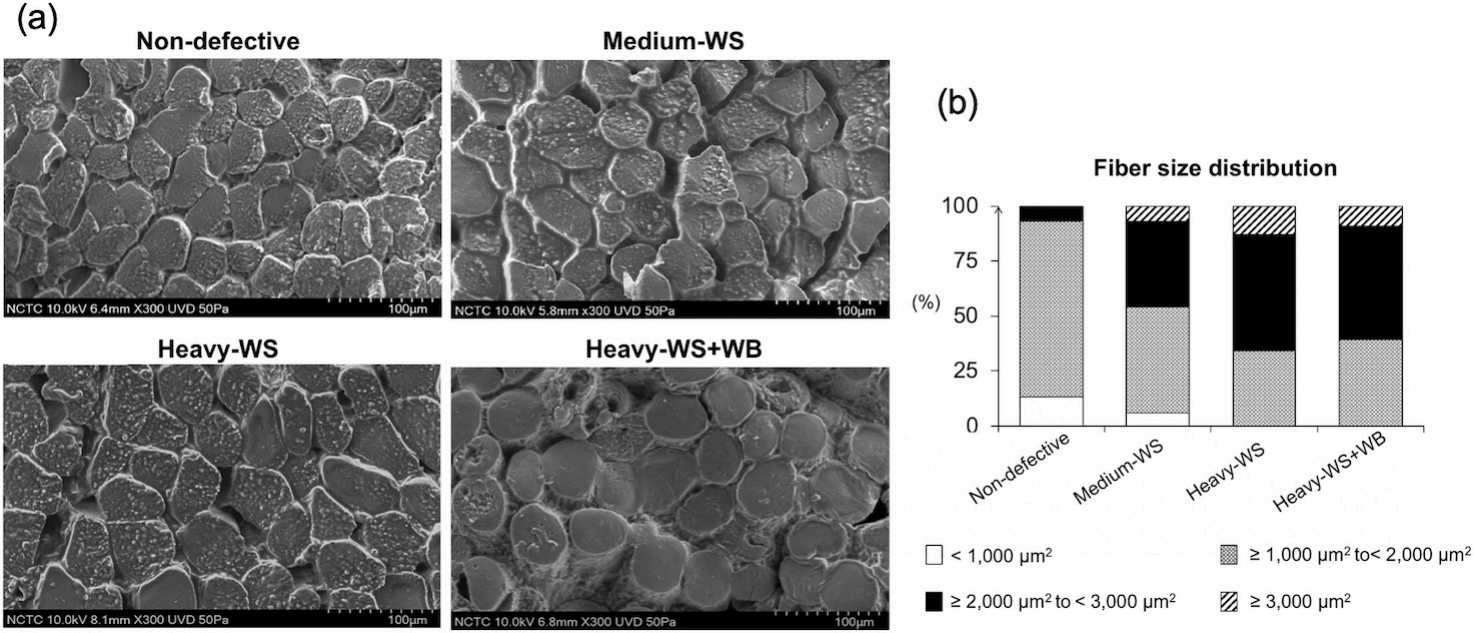
Characteristics of muscle fibers of breast meat. The samples collected from 6-week-old male Ross 308 broilers classified into four groups based on the degree of white striping (WS) and wooden breast (WB) abnormalities combined with carcass weight; “non-defective” = non-defective (n = 4), “medium-WS” = WS-affected samples with carcass weight ≤ 2.5 kg (n = 7), “heavy-WS” = WS-affected samples with carcass weight ≥ 2.5 kg (n = 6), and “heavy-WS+WB” = WS and WB affected samples with carcass weight ≥ 2.5 kg (n = 3). (a) Microscopic images of cross-sectional pectoralis major muscle fibers (300× magnification) displayed under field-emission scanning electron microscope using an acceleration voltage of 10 kV. Scale bar = 100 μm. (b) Bars illustrate size distribution (%) of muscle fiber regarding cross-sectional area within each sample group.

In this study, the fiber size distribution of the samples has been determined using an FE-SEM (Fig 1A). The muscle fibers of non-defective meat were composed mainly of fibers with cross-sectional area between ≥ 1,000 to < 2,000 μm^2^ (Fig 1B). None of the fibers found in non-defective samples were larger than 3,000 μm^2^. In the abnormal samples, the sizes of muscle fibers tend to be larger. Particularly, fibers with area < 1,000 μm^2^ were not found in the heavy samples. The present observation was consistent with previous reports of Petracci et al. [44], Clark and Velleman [25] and Daughtry et al. [45].

Development of WS and WB myopathies have been widely hypothesized to be associated with selection pressure for accelerated growth rate and enlarged breast muscle [3, 22]. The massive muscle fibers reduce perimysial space available for capillaries while increasing the ratio of muscle fiber volume to capillary volume. The increased size of muscle fibers increases the distance for oxygen diffusion and waste removal, thereby creating oxidative stress and eventually myodegeneration [25]. In a recent study of Sihvo et al. [46], microscopic morphometry of pectoral blood vessels within the WB samples was examined. The transverse myofiber area per blood vessel number observed in the unaffected area was greater than that from the area exhibiting the WB lesion. Their findings evidenced the relative decrease in blood supply associated with WB development, suggesting the potential development of hypoxia within the abnormal samples.

In the previous study of Daughtry et al. [45], characteristics of satellite cells in pectoralis major muscle were compared between the six smallest and the six largest broilers raised under similar procedure and slaughtered at the age of 5 or 8 weeks [45]. As expected, at the same age, the six largest birds comprised a greater number of hypertrophied muscles in accompanied with an incidence of WS lesion. In addition, a declined number of satellite cells along with diminished capabilities of proliferation and differentiation were observed among those hypertrophied muscles compared to those of small birds. Collectively, the altered satellite cells may account in part for impeding muscle regeneration in the WS- and WB-affected birds.

### Expression of *HIF1A* in the myopathic skeletal muscle

**Fig 2:**
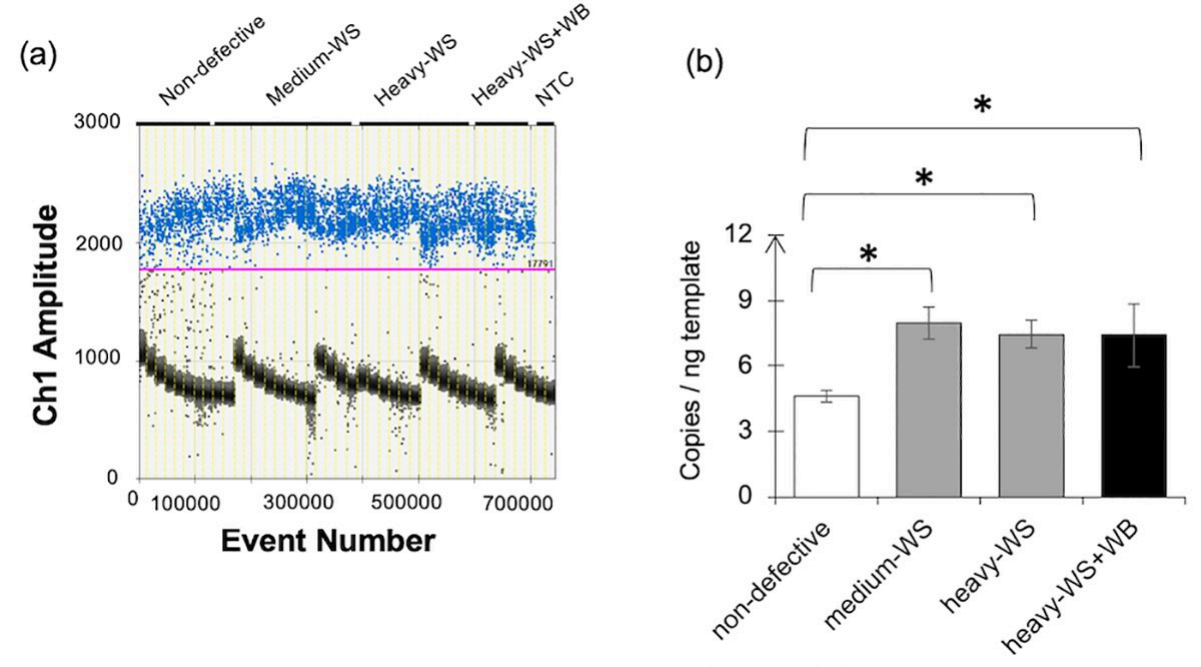
Absolute expression of hypoxia-inducible factor 1 alpha subunit (*HIF1A*) gene. The absolute transcript abundance was determined using an EVAGREEN-based droplet digital polymerase chain reaction. (a) One-dimensional plots between fluorescent intensity and droplet events. Positive (high amplitude) and negative (low amplitude) droplets are manually separated by threshold line. (b) Absolute expression level of the gene evaluated in 6-week-old male Ross 308 broilers classified into four groups based on the degree of white striping (WS) and wooden breast (WB) abnormalities combined with carcass weight; “non-defective” = non-defective (n = 4), “medium-WS” = WS-affected samples with carcass weight ≤ 2.5 kg (n = 7), “heavy-WS” = WS-affected samples with carcass weight ≥ 2.5 kg (n = 6), and “heavy-WS+WB” = WS and WB affected samples with carcass weight ≥ 2.5 kg (n = 3). Bars represent mean ± standard error in copies per nanogram cDNA template. Asterisks above each bracket indicate statistical difference between each pair. *p<0.05, **p<0.01, ***p<0.001, NTC = no template control.

Absolute transcript abundances of *HIF1A* determined using an EVAGREEN-ddPCR platform were illustrated in the plot of fluorescence intensity of the droplets (Fig 2A). As depicted in Fig 2B, *HIF1A* abundance significantly increased from 4.6 ± 0.3 copies per ng cDNA template in non-defective skeletal muscle samples to 8.0 ± 0.7, 7.5 ± 0.6, and 7.4 ± 1.4 copies/ng in medium-WS, heavy-WS, and heavy-WS+WB respectively (p<0.05). The current ddPCR results for *HIF1A* transcript abundance are consistent with the previous transcriptome profile of breast muscle with WS condition [27]. There were no significant effects of carcass weight on *HIF1A* abundance.

It is generally known that HIF-1 dynamically regulates glucose metabolism by activating anaerobic glycolysis while reducing oxidative respiration when the cells experience hypoxia [33]. This metabolic switch is maintained to minimize the risk of energy depletion as well as excessive ROS accumulation [33]. In addition to its regulatory role on metabolism, an association between HIF-1α and muscle development and regeneration has been addressed [47, 48]. Majmundar et al. [47] reported that suppression of *HIF1A* enhanced skeletal muscle regeneration in muscle cells derived from adult mice, suggesting that HIF-1 negatively regulates adult muscle regeneration after ischemic injury. Yang et al. [48] developed a mouse model of which *HIF1A* and *HIF2A* were specifically co-deleted only from its satellite cells, the myogenic adult stem cells. Upon exposure to an ischemic injury, the knockout mice exhibited reduced numbers of satellite cells as well as a smaller regenerative area relative to that of wild-type. Their findings indicate that the deficiency of *HIF1A* and *HIF2A* delayed muscle regeneration by reducing number of satellite cells. Recently, the effects of hypoxic preconditioning on myogenesis were addressed in C2C12 mouse myoblasts [49]. After 24-h preconditioning at 1% oxygen, HIF-1α abundance in nucleus was increased, indicating that HIF-1α was activated. The hypoxic preconditioning also promoted myoblast differentiation and formation of hypertrophic myotubes to differentiate under normal oxygen concentration.

In this study, to ascertain whether the increased *HIF1A* was a cause or a consequence of the myopathies, further investigation is required. Nevertheless, the increased transcript abundance of *HIF1A* in our study not only supports the hypothesis that development of hypoxia within the muscle of is one of the underlying factors in development of WS and particularly WB abnormalities but could be indicative of satellite cell activity of muscle regeneration.

### Transcriptional levels of the stress-response genes

**Fig 3:**
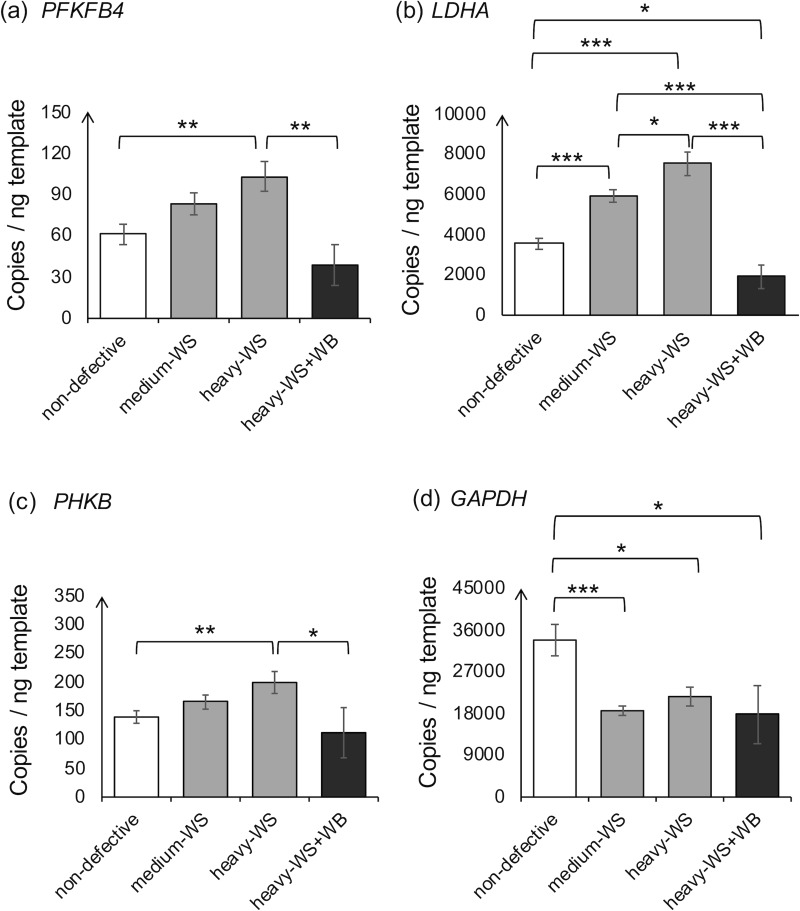
Absolute transcript abundances of genes associated with glucose utilization. The genes include (a) 6-phosphofructo-2-kinase/fructose-2,6-biphosphatase 4 (*PFKFB4*), (b) lactate dehydrogenase kinase subunit A (*LDHA*), (c) phosphorylase kinase beta subunit (*PHKB*), and (d) glyceraldehyde-3-phosphate dehydrogenase (*GAPDH*). The absolute expression level of the target genes was quantified in 6-week-old male Ross 308 broilers classified into four groups based on the degree of white striping (WS) and wooden breast (WB) abnormalities combined with carcass weight; “non-defective” = non-defective (n = 4), “medium-WS” = WS-affected samples with carcass weight ≤ 2.5 kg (n = 7), “heavy-WS” = WS-affected samples with carcass weight ≥ 2.5 kg (n = 6), and “heavy-WS+WB” = WS and WB affected samples with carcass weight ≥ 2.5 kg (n = 3). Bars represent mean ± standard error in copies per nanogram cDNA template. Asterisks above each bracket indicate statistical difference between each pair. *p<0.05, **p<0.01, ***p<0.001.

Upon exposure to hypoxic environments, cellular activities are reprogrammed to maintain homeostasis, hence cell survival. Under this condition, HIF-1 can affect transcription of genes involved in glycolysis and gluconeogenesis [33]. Herein, expressions of *PFKFB4*, *LDHA*, *PHKB*, and *GAPDH* were determined. The transcript abundances of *PFKFB4*, *LHDA*, and *PHKB* exhibited similar trends, increasing in the WS affected samples compared to those of non-defective ones (p<0.05); however, their expressions decreased as WB developed (p<0.05) (Fig 3A to 3C). Conversely, the greatest expression of *GAPDH* was observed in the non-defective samples (p<0.05) compared to the defective samples and there were no significant differences in *GAPDH* abundances among the abnormal samples (Fig 3D).

The *PFKFB4* encodes a bifunctional kinase/phosphatase enzyme that regulates glucose flux through the synthesis and degradation of fructose-2,6-bisphosphate concentration [50, 51]. Overexpression of *PFKFB4* via the HIF-1 dependent mechanism has been identified in several cancer cell types, e.g. human prostate cancer, human hepatoma, gastric, colon, lung, and breast malignant tumors as an adaptive metabolism in a hypoxic environment [52, 53, 54]. A recent study of Houddane et al. [54] demonstrated that *PFKFB4-*knockdown in Jurkat E6-1 cells (human T lymphocytes) resulted in reductions in cell proliferation, protein synthesis, and lactate accumulation, highlighting the crucial roles of this gene in coupling glycolysis to cell proliferation and protein synthesis. In the present study, the increased average *PFKFB4* transcript abundances in the heavy-WS samples (102 copies/ng) compared to that of the non-defective samples (61 copies/ng) suggests that the cellular response is an attempt to increase glucose uptake and glycolytic flux under the limited oxygenation [51].

The *LDHA* transcript was highly expressed in the heavy-WS samples (7,533 copies/ng), followed by the medium-WS (5,921 copies/ng) and non-defective (3,552 copies/ng) but substantially reduced in the heavy-WS+WB samples (1,924 copies/ng). The results were consistent with the previous studies, reporting an activated expression of *LDHA* under an experimentally hypoxic condition [55, 56] primarily through the induction of HIF-1 [55, 57]. The *LHDA-*encoding enzyme regenerates the oxidized form of nicotinamide adenine dinucleotide as the supply for glycolysis.

The abundance of *PHKB* in heavy-WS was approximately 199 copies/ng which was greater than that of non-defective (138 copies/ng) and heavy-WS+WB (110 copies/ng) samples but did not significantly differ from that of medium-WS (164 copies/ng). The *PHKB* gene encodes the regulatory subunit of phosphorylase kinase which catalyzes glycogen breakdown cascade in response to various signals to assure the continuous energy availability for cellular activities [58]. The increased average *PHKB* abundance in the heavy-WS samples implies the increased energy requirement of the muscle cells under hypoxia [59].

The decreases in *PFKFB4*, *LDHA*, and *PHKB* abundances within the heavy-WS+WB samples might be related to a reduced cellular glycolysis in agreement with a previous observation in which reduced levels of glycolytic intermediates pyruvate and lactate within the WB muscle were observed [41]. Moreover, proteomic profiling for enzymes and sarcoplasmic proteins indicated that glycolysis and gluconeogenesis were the two major down-regulated pathways within the breast muscle exhibiting severe WS along with WB myopathies [28]. Unlike the heavy-WS+WB samples, *PFKFB4*, *LDHA*, and *PHKB* abundances in the medium-WS and heavy-WS samples markedly increased, suggesting divergent metabolic mechanisms within the muscle between WS and WB myopathies.

To elucidate further the etiologies of these syndromes, the lactate content and pH of the samples were determined at both 20-min and 24-h postmortem (Table 2). The results indicated no difference in Lac_20m_ among all muscle samples whereas pH_20m_ of the heavy-WS and heavy-WS+WB were slightly greater than that of non-defective samples. Subsequently, in the post-rigor period, the abnormal meat samples showed comparable Lac_24h_ but these values were lower than that of the non-defective group (p<0.05). The trend of pH_24h_ was in accordance with that of the Lac_24h_. An aberrantly high ultimate pH was consistently reported as associated with development of WS and WB abnormalities [12, 13, 20, 26, 60, 61]. Significant decreases in Gly_20min_ were observed within the abnormal samples compared to non-defective group (Table 3). The discrepancy regarding the activation of gene expression and reduced abundance of the respective metabolites is still not fully comprehended. Considering gene expression aspect, by employing microarray technique, Marchesi et al. [27] identified a down-regulation of *LDHA* whereas the gene encoding lactate dehydrogenase B (*LDHB*) was up-regulated in WS-affected chicken muscle compared with those in non-defective ones. At protein level, a decreased LDH along with a reduced glycolytic potential and increased ultimate pH in WS and WB birds have been addressed [28, 41]. Alnahhas et al. [61] compared WS incidence between two divergent broiler lines selected for high ultimate pH (pH 6.11 to 6.13) and low ultimate pH (pH 5.66 to 5.72). They reported a higher WS incidence and severity in the lines with high ultimate pH. In the study of Kuttappan et al. [62], an elevated serum LDH level in commercial broilers exhibiting severe WS compared with that of unaffected birds was detected, indicating a leakage of LDH from the degenerative muscle fibers into serum.

**Table 2:** The pH values and lactate content in the breast samples of 6-week-old broilers^1^.

Properties	Non-defective	Medium-WS	Heavy-WS	Heavy-WS+WB
	(n = 4)	(n = 7)	(n = 6)	(n = 3)
**Skeletal muscle sample**				
Lac_20m_ (mg/g)	2.61 ± 0.09	2.61 ± 0.12	2.68 ± 0.40	2.33 ± 0.36
pH_20m_	6.79^b^ ± 0.05	6.86^ab^ ± 0.02	6.96^a^ ± 0.03	6.93^a^ ± 0.03
**Meat sample**				
Lac_24h_ (mg/g)	9.11^a^ ± 0.67	6.00^b^ ± 0.31	6.23^b^ ± 0.33	5.93^b^ ± 0.77
pH_24h_	5.72^b^ ± 0.10	5.93^ab^ ± 0.06	5.98^a^ ± 0.05	5.99^a^ ± 0.06

^1^ Data are presented as mean ± standard error. Superscripts indicate statistical difference among non-defective and WS with different carcass weight (p<0.05). pH_20m_ and pH_24h_ = pH values determined from 20-min postmortem muscle, and 24-h postmortem meat samples, respectively. Lac_20m_ and Lac_24h_ = lactate content determined from 20-min postmortem muscle, and 24-h postmortem meat samples, respectively.

**Table 3:** Glycogen and ATP status determined in the 20-min postmortem pectoralis major muscle of 6-week-old broilers^1^.

Properties	Non-defective	Medium-WS	Heavy-WS	Heavy-WS+WB
	(n = 4)	(n = 7)	(n = 6)	(n = 3)
Glycogen (mg/g)	7.12^a^ ± 1.32	1.43^b^ ± 0.62	1.42^b^ ± 0.75	1.47^b^ ± 0.14
R-value (A_250_/A_260_)	0.81^a^ ± 0.01	0.76^b^ ± 0.02	0.70^c^ ± 0.01	0.71^c^ ± 0.01

^1^ Data are presented as mean ± standard error. Superscripts indicate statistical difference among non-defective and WS with different carcass weight (p<0.05). R-value = ratio of absorbance at 250 nm to absorbance 260 nm.

The lower glycogen content in defective meat was previously observed in WB meat reported by Abasht et al. [41]. Berri et al. [63] proposed that the enlarged muscle fiber size might result in decreased glycogen content in muscle and increased susceptibility to WS. Considering the R-values (Table 3), the heavy-WS and heavy-WS+WB samples exhibited the lowest R-values whereas the medium-WS showed the intermediate value between those and non-defective group. This absorbance ratio has been used to estimate postmortem ATP status within the muscle tissue [64]. The low R-values in the heavy broilers reflect a low concentration of inosine nucleotides relative to concentration of adenosine nucleotides within the muscle samples, hence suggesting a less ATP depletion within those defective heavy birds.

Based on our findings, it could be speculated that even though transcriptions of genes involved in glycolytic pathway within the moderate WS-affected muscle cells are more likely induced through HIF-1 in response to the hypoxic state, the formation of lactate could be limited due to the loss of LDH from the abnormal muscle fibers. Ones cannot exclude the possibilities that the reduced LDH in the WS muscle may be because of the leakage of the enzyme during myodegeneration [62] or the limited glycogen stored within the abnormal muscles [63, 65]. A better comprehension regarding the diverse metabolic mediation at different WS and WB severity remains to be established.

Reduced transcript abundances of *GAPDH* from 33,940 copies/ng in non-defective samples to approximately 20,000 copies/ng in the abnormal samples (p<0.05) were quite unexpected as transcription of this gene is usually induced by HIF-1 signaling [66]. The primary function of *GAPDH*-encoding enzyme is to activate conversion of glyceraldehyde 3-phosphate to D-glycerate 1,3-bisphosphate in the glycolytic pathway for energy production. Later, additional functions of GAPDH distinct from energy production have been reported. Decreased GAPDH protein level has been shown to induce cell cycle arrest and hinder cell proliferation in carcinoma cell lines, suggesting a regulatory role [67]. The protective effect of GAPDH against oxidative-stress-induced apoptosis has been demonstrated in vascular smooth muscle cells [68]. Taken together, down-regulation of *GAPDH* within the WS and WB samples may link to other cellular roles of GAPDH in response to the hypoxic condition within the muscle.

### Expression of genes encoding glutathione S-transferases

**Fig 4:**
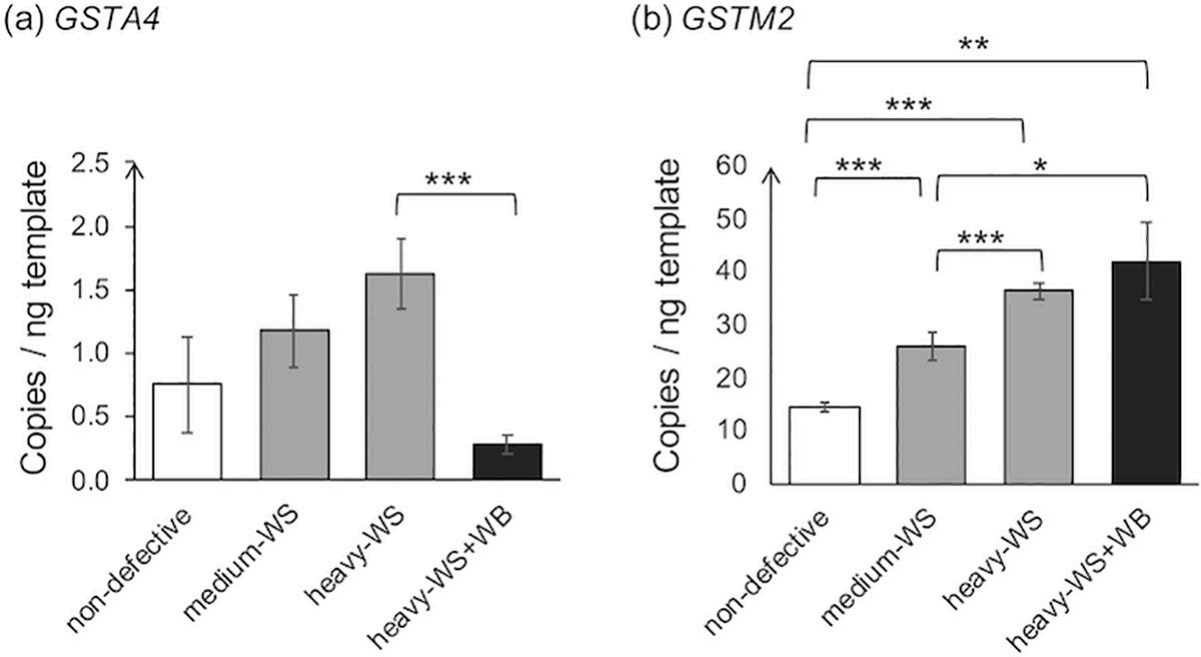
Absolute transcription levels of genes encoding glutathione S-transferases. The absolute expression level of the target genes, including (a) glutathione S-transferase alpha 4 (*GSTA4*) and (b) glutathione S-transferase mu 2 (*GSTM2*), was quantified in 6-week-old male Ross 308 broilers classified into four groups based on the degree of white striping (WS) and wooden breast (WB) abnormalities combined with carcass weight; “non-defective” = non-defective (n = 4), “medium-WS” = WS-affected samples with carcass weight ≤ 2.5 kg (n = 7), “heavy-WS” = WS-affected samples with carcass weight ≥ 2.5 kg (n = 6), and “heavy-WS+WB” = WS and WB affected samples with carcass weight ≥ 2.5 kg (n = 3). Bars represent mean ± standard error in copies per nanogram cDNA template. Asterisks above each bracket indicate statistical difference between each pair. *p<0.05, **p<0.01, ***p<0.001.

Under hypoxic conditions, formation of ROS induces oxidation of cellular components, including proteins, lipids and DNA, triggering cell damage [69]. One mechanism of removing the harmful products is via the glutathione detoxification pathway [70]. The GST superfamily is responsible for catalyzing conjugation of glutathione and detrimental electrophiles into less toxic forms that are available for subsequent detoxification steps [37, 71]. In this study, as shown in Fig 4, gene expression of two GST classes, including *GSTA4* and *GSTM2*, were focused.

Despite no significant difference in *GSTA4* abundance among non-defective (0.8 copy/ng) and the WS-affected groups (1.2 and 1.6 copies/ng in medium-WS and heavy-WS, respectively), there was a substantial decrease in *GSTA4* level when WB developed (0.28 copies/ng). A low *GSTA4* mRNA abundance in the current chicken skeletal muscle was as expected as the alpha GST class is not a major GST in skeletal muscle [72]. Nevertheless, its essential activities in skeletal muscle have been documented in a previous study of Apidianakis et al. [73] in which *GSTA4* mRNA level was nearly 2-fold down-regulated in human and mouse skeletal muscle tissues subjected to a severe thermal injury. Growth of human hepatoma HepG2 cells either transfected with mouse *GSTA4* cDNA or an insert-free vector was determined under oxidative stress. An increased viability of the *GSTA4-*transfected cells was observed after the cells were exposed to H_2_O_2_ or organic hydroperoxides. In addition, when concentration of cumene hydroperoxides, one of the testing oxidative stress inducers, was increased from 0 μM to 6 μM, proliferation of both cells decreased but the *GSTA4*-HepG2 cells showed a slower decreasing rate [74].

For *GSTM2*, the expression of this gene increased significantly as the myopathies advanced. The heavier the birds, the greater the *GSTM2* was expressed (p<0.05). In addition, absolute abundance of this gene was approximately 10-fold higher than those of *GSTA4*. The findings suggested the major role of *GSTM2* isoform in detoxification of oxidative stress associated with WS and WB myopathies. Li et al. [37] found that increased *GSTM2* expression was associated with inhibition of oxidative stress-induced cell apoptosis and inflammation in mouse kidneys. Additionally, the protective activity of GSTM2 enzyme against aminochrome, the toxic product of dopamine oxidation, has been shown in astrocytes [75].

Collectively, the altered expression of *GSTA4* and *GSTM2* affirmed the defensive mechanisms of the muscle cells against increased oxidative stress in the abnormal muscle. Further experiments must be carried out to characterize the different responses between those two genes in association with development of WS and WB myopathies, particularly the decreased *GSTA4* in the WB-affected muscle. It is worth noting that the cellular defensive mechanisms against oxidative stress require numbers of hierarchical enzyme systems which can become saturated at a certain threshold of the oxidative stress [74]. This may be a partial manifestation of the decreased *GSTA4* in the severely injured WB-affected sample.

### Expression of genes associated with muscle regeneration

Hypoxic stress has been recognized as one of the major causes of muscle injury [38]. Previous histological studies commonly addressed degenerative lesions among the breasts affected with WS and WB abnormalities [6, 10, 13, 25, 60, 76]. Based on our recent report [13], deposition of adipocytes, nuclear internalization, accumulation of macrophages, thickened endomysial and perimysial connective tissue layers and necrotic fibers were observed in the muscle exhibiting either WS alone or in accompanied with WB lesion.

**Fig 5:**
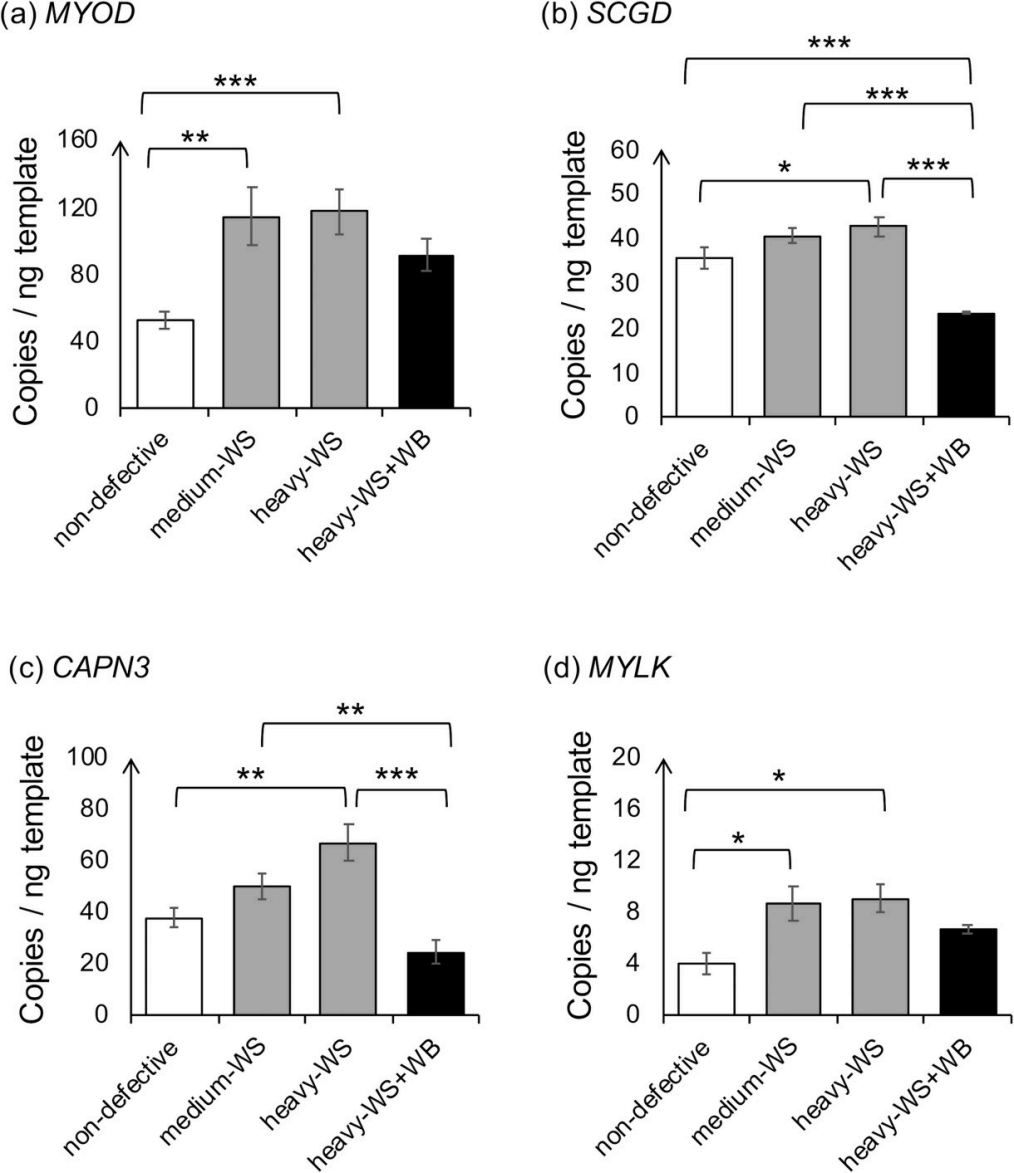
Absolute transcription levels of genes associated with muscle regeneration. The absolute expression level of the target genes, including (a) myogenic differentiation 1 (*MYOD1*), (b) sarcoglycan delta subunit (*SCGD*), (c) calpain 3 (*CAPN3*) and (d) myosin light chain kinase (*MYLK*), was quantified in 6-week-old male Ross 308 broilers classified into four groups based on the degree of white striping (WS) and wooden breast (WB) abnormalities combined with carcass weight; “non-defective” = non-defective (n = 4), “medium-WS” = WS-affected samples with carcass weight ≤ 2.5 kg (n = 7), “heavy-WS” = WS-affected samples with carcass weight ≥ 2.5 kg (n = 6), and “heavy-WS+WB” = WS and WB affected samples with carcass weight ≥ 2.5 kg (n = 3). Bars represent mean ± standard error in copies per nanogram cDNA template. Asterisks above each bracket indicate statistical difference between each pair. *p<0.05, **p<0.01, ***p<0.001.

Following degeneration, the cascade of repair processes is activated. The essential events include orchestration of damaged cell removal and formation of new myofibers [38]. Various enzymes are simultaneously activated to initiate sarcolemma repair [77]. Among those, expressions of the genes encoding proteins participating in those repair-responsive pathways, *MYOD1*, *SCGD*, *CAPN3* and *MYLK*, were analyzed in this experiment (Fig 5A to 5D). Similar changes in transcript abundance were observed among those four genes. Compared with non-defective samples, the heavy-WS exhibited the significant increases in *MYOD1*, *SCGD*, *CAPN3* and *MYLK* (p<0.05). Absolute transcripts of *CAPN3* and *SCGD* decreased when the WS was in accompanied with WB lesion.

Muscle regeneration after injury has a similar process as muscle development during embryogenesis [38]. The regeneration of damaged muscle occurs by activation of the satellite cell regeneration process, which is characterized by high expression of myogenic regulatory factors (MRFs) [78, 79]. Among those MRFs, *MYOD1* encodes myoblast determination protein which regulates muscle differentiation. Increased expression of this gene was previously observed in satellite cells of fast-growing turkeys in comparison to those of the slow-growing birds [80]. Recently, Velleman and Clark [22] reported an increase in *MYOD* abundance in WB-affected broiler breast muscle compared with non-WB but this change in *MYOD* expression was breed-dependent. In the present study, absolute expression *MYOD1* was significantly up-regulated from 52 copies/ng in non-defective to 115 copies/ng and 118 copies/ng in medium-WS and heavy-WS, respectively which was consistent with the observation of Velleman and Clark [22]. For heavy-WS+WB sample group, although its expression level did not show statistical increase compared to that of non-defective, its absolute expression was reported at 91 copies/ng. The insignificant difference of *MYOD1* transcripts could be the effect of large variation combined with small number of the heavy-WS+WB sample set.

The *CAPN3* gene encodes the muscle-specific calcium-dependent protease located in the sarcomere. The function and activation mechanisms of CAPN3 are not fully understood but it has been identified as a protease involved in the muscle regeneration process via promotion of satellite cell formation [81, 82]. The CAPN3 enzyme catalyzes degradation of damaged skeletal muscle proteins into smaller peptides for cellular removal processes [83]. By conducting a genome-wide association study for WS and meat quality traits, Pampouille et al. [84] identified *CAPN3* as one of the four candidate genes located in the pleiotropic region strongly associated with water holding capacity and color of chicken breast meat. Based on the study of Pampouille et al. [84], *CAPN3* may influence such meat quality traits by governing muscle composition and structure. In the current study, absolute abundance of *CAPN3* was up-regulated in heavy-WS (67 copies/ng) in comparison to that of non-defective samples (38 copies/ng). An increased *CAPN3* transcription implies the repairing attempt of moderately damaged WS-affected muscle via the activation of satellite cells. However, compared to heavy-WS, *CAPN3* expression in heavy-WS+WB (24.6 copies/ng) was reduced. Further investigations regarding the reduction of *CAPN3* when WS was accompanied with WB lesion or the missing activation of *CAPN3* compared with non-defective samples must be established to obtain better understanding in this aspect.

Expression of *SCGD* increased in heavy-WS (43 copies/ng) compared to that of non-defective samples (36 copies/ng) but significantly (p<0.05) decreased in heavy-WS+WB (23 copies/ng). Sarcoglycan complex, comprising four subunits alpha-, beta-, delta- and gamma, binds with dystrophin-associated glycoprotein complex and plays a significant role in maintaining the link between the actin cytoskeleton and the extracellular matrix [85]. Disruption of this protein complex is associated with an impaired muscle membrane integrity. In mice, *SCGD*-knockout skeletal muscle developed oxidative stress, fibrosis as well as impaired activities of various enzymes required for countering cellular oxidative stress [86]. In addition to membrane stabilization, the sarcoglycan complex regulates glucose homeostasis [87]. In the previous study of Solares-Pérez et al. [88], the muscle fibers of *SCGD*-knockout mice exhibited a decrease in the amplitude of intracellular Ca^2+^ signal transients, ultimately inducing muscular dystrophy. Additionally, suppression of *SCGD* expression contributed to development of necrosis, fatty infiltration and membrane fragility in the muscle fibers of *SCGD*-knockout mice [89]. Decreased abundance level of α-sarcoglycan was observed in turkey skeletal muscle with the pale, soft and exudative (PSE) defect which exhibited abnormal regulation of cellular glucose breakdown [90]. An association between delta-sarcoglycan protein and tissue regeneration in mice was also reported by Straub et al. [91] and Ramirez-Sanchez et al. [86]. The studies of Hack et al. [92] and Zhu et al. [93] suggest that sarcoglycan complex functions as a unit. Loss of beta subunits alone sufficiently reduced abundances of the other sarcoglycan subunits to cause progressive muscle dystrophy in mice [94]. In this study, the increased expression of *SCGD* in the heavy-WS muscle samples might reflect the attempt of the damaged muscle cells to repair and maintain the sacrolemma integrity as well as to encounter oxidative stress [95]. On the other hand, the suppression of *SCGD* in heavy-WS+WB group suggests the altered disposition of sarcoglycan molecule as delta-sarcoglycan plays a central role as the assembly core for the sarcoglycan [86]. Along with the other genes, the reduced expression of *SCGD* potentially led to the severe atrophic fibrosis as previously observed in the *SCGD*-knockout mouse skeletal muscle [86].

Myosin light chain kinases (MYLK) are Ca^2+^/calmodulin-regulated enzymes that catalyze phosphorylation of the regulatory light chains of myosin. In chickens, skeletal-muscle specific MYLK (skMYLK) and the smooth-muscle specific isoform (smMYLK) have been identified and characterized [96]. In this study, the abundance of *MYLK*, the gene encoding smMYLK, increased from 4 copies/ng in non-defective to 9 copies/ng in medium-WS and heavy-WS samples (p<0.05). The heavy-WS+WB samples showed intermediate *MYLK* expression (7 copies/ng) which was not statistically different from those of either non-defective or the samples exhibiting WS alone. Unlike skMYLK which is expressed exclusively in skeletal muscle, the smMYLK is widely found in all adult tissues, including skeletal muscle [97]. The presence of smMYLK at low levels in skeletal muscle has been demonstrated in differentiating skeletal muscle and is crucial for regulating sarcomere organization [98, 99]. Additionally, in human pulmonary vein endothelial cell lines, MYLK protein level was up-regulated through the mediation of HIF-1α with concomitant vascular endothelial barrier dysfunction when the cells were exposed to hypoxic stress [100]. The dysregulated barrier properties of the vascular endothelial cells, the cells that line the interior surfaces of blood vessels, have been evidenced to interfere with the balance between intravascular and extravascular compartments, leading to damages ranging from a small and reversible injury to an irreversible necrosis [101]. Accordingly, the increased smooth muscle-specific *MYLK* in the present WS-affected samples not only suggests that the muscle cells of WS-affected samples were in differentiating stage, but also supports the event of hypoxia in the WS samples and the potential impaired vascular endothelial function in the abnormal muscle.

According to the current ddPCR results, it seems that expression of *HIF1A*, *GAPDH*, and *GSTM2* in the WS and WS+WB muscle samples changes in a similar trend. The up-regulation of *HIF1A* in accordance with the presence of large muscle fibers strongly supports the potential hypoxic state within the defective samples. Interestingly, the expression patterns of the other focused genes were different between WS and WS+WB groups. Most of them exhibited the reduction in heavy-WS+WB in comparison with the heavy-WS group. The decreased mRNA abundance of those genes in the muscle affected with WB lesion may partly reflect a global mRNA degradation occurring during apoptotic [102] and necrotic cell death [103]. The findings herein suggest the more deleterious event resulting in a greater cell death in WB abnormality. The diverse responsive mechanisms at molecular level towards such stress could be anticipated and these could lead to the distinct appearances between WS and WB.

Based on the study of Radaelli et al. [104] considering the WS and WB myopathies among the broilers slaughtered at different ages, the WB abnormality was present only in the chickens slaughtered at 46 days of age whereas WS condition was first detected among the 14-day-old birds and the lesion became more severe with age. Our recent logistic regression analysis revealed an increased likelihood of WB occurrence associated only with breast yield but the severity of WS can be linked with age and breast yield [13]. Upon examining pectoral microvessel density of pectoralis major muscle collected from broilers with or without WB myopathy under a light microscope, Sihvo et al. [46] described a significantly reduced number of blood vessels in muscles with focal WB among the birds slaughtered at the age of 18 and 24 days; however, there was no difference among the birds with the age of 35 or 38 days. In addition to the reduced blood vessel number, the greatest fiber area per number of blood vessels was addressed in the unaffected area of tissue collected from the birds with focal WB. Together, the speculation is that, in the WS-affected group, transcriptions of those genes responsible for glucose utilization, ROS detoxification and muscle regeneration was provoked by HIF-1 in response to the hypoxia [105]. The WB lesion, however, seems to be a consequence of a chronic myodegeneration potentially from a long exposure to severe hypoxia. Our speculation was supported by the recently published RNA-sequencing report of Papah et al. [106] in which gene expression profiles of pectoralis muscle biopsies collected from high-breast-yield broilers during the age of 2 to 4 weeks were compared between WB-affected and unaffected samples. Their functional pathway analysis suggested that although WB characteristics was undetectable in the early development of the WB-affected birds, the molecular perturbations, particularly those involved in vascular pathology and metabolic dysregulation, had already occurred.

In summary, we reported altered expression of several candidate genes correlated with development of WS and WB myopathies, which would be essential for further elucidating the underlying mechanisms that link to the development of such abnormalities. By quantifying absolute expression, it was found that *HIF1A* were expressed somewhat low across all abnormality levels but significantly increased for approximately 1.7-fold in the abnormal samples. The differential expression of *HIF1A* coupled with the other focused genes suggests a potent hypoxic state and the molecular activities in response to the oxidative stress within the muscles of broilers exhibiting WS and WB abnormalities. The processes of muscle regeneration were, however, appeared to be impeded in the abnormal muscles. Different expression patterns of the selected genes between WS and WB samples, particularly the markedly reduced mRNA abundances in the muscle affected with WB abnormality, suggest the pronounced detrimental effects of WB and the divergent responsive mechanisms at transcriptional level that contribute to the distinguishing macroscopic characteristics between those myopathies. Although the root cause of WS and WB disorder are still under investigation, our findings suggest that an enlarged muscle fiber size was more likely the shared foundation triggering oxidative stress that induces WS and WB myopathies in the breasts of commercial broilers.
